# What Types of Educational Practices Impact School Burnout Levels in Adolescents?

**DOI:** 10.3390/ijerph17041152

**Published:** 2020-02-12

**Authors:** Nicolas Meylan, Joël Meylan, Mercedes Rodriguez, Patrick Bonvin, Eric Tardif

**Affiliations:** UER Développement de l’enfant à l’adulte University of Teacher Education, HEP Vaud, 1007 Lausanne, Switzerland; Joel.meylan@hepl.ch (J.M.); Mercedes.rodriguez@hepl.ch (M.R.); patrick.bonvin@hepl.ch (P.B.); eric.tardif@hepl.ch (E.T.)

**Keywords:** educational practices, school burnout, adolescents, teachers

## Abstract

This study explores the relationship between educational practices perceived by high school students and their level of burnout, as defined by emotional exhaustion, cynicism and inadequacy. A total of 287 adolescents (146 girls) aged between 14 and 19 years old (*M* = 16.08, *SD* = 1.01) and recruited from a public high school in French-speaking Switzerland completed a questionnaire regarding perceived educational practices and school burnout. Results from path analysis showed that the three dimensions of burnout were negatively associated with certain teacher- and school-related educational practices. More precisely, support for struggling students (ß = −0.24, *p* < 0.001) as well as teaching time (ß = −0.16, *p* < 0.05) were predictors of exhaustion (*R*^2^ = 0.27). Teachers’ instructional behavior (ß = −0.22, *p* < 0.01) and teacher motivation (ß = −0.31, *p* < 0.001) were predictors of cynicism (*R*^2^ = 0.20) and application of rules (ß = −0.21, *p* < 0.01) predicted inadequacy (*R*^2^ = 0.09). These educational practices should be of particular interest when it comes to strengthening the protective role of schools and teachers against school burnout in adolescents.

## 1. Introduction

As places of socialization and development, schools hold considerable importance in adolescents’ lives [1,2]. They spend a lot of their time in school, where their main activity can be likened to a job in which one must satisfy a number of requirements: class attendance, taking courses and exams, and obtaining degrees and certifications [3]. The demands associated with school requirements contribute to intellectual and social–emotional development as they convey both structure and gratification. However, they can also lead to difficulties for some adolescents [4,5]. Indeed, increases in pressure for success and performance in today’s society may tax the individual’s resources and may lead to stress-induced suffering in adults at work as well as adolescents in schools [6,7].

International surveys have shown that both school related stress and health problems associated with stress have risen significantly in adolescents [8,9,10]. A chronic exposure to academic stress can result in school burnout, defined as an emotional state of exhaustion, cynicism and depersonalization [7]. Describing the impact of school demands during adolescence in terms of burnout has drawn growing interest [11,12,13], partly because school difficulties experienced by adolescents are one of the most common motives for seeking help from health professionals [14].

### 1.1. School Burnout

The concept of burnout, initially studied in adults within their professional contexts, including teaching [15], has recently been increasingly applied to the experience of teenage students in schools. According to Salmela-Aro, Kiuru, Leskinen, and Nurmi [16], school burnout can be considered as a response to chronic school stress and is characterized by (1) exhaustion, (2) cynicism and (3) a feeling of inadequacy. Exhaustion refers to the feeling of being overwhelmed by school-related pressure, including chronic fatigue due to concerns and ruminations about schoolwork. Cynicism towards school refers to a detached or indifferent attitude towards school in general, with a loss of interest and motivation in schoolwork and an inability to make sense of it. A feeling of inadequacy as a pupil refers to the feeling of “not being up to the mark” in schoolwork, a lack of achievement in schoolwork and at school in general.

The prevalence of school burnout varies according to burnout definitions, study quality and assessments methods. For instance, it has been shown that around 6.8% of Slovenian adolescents display a worrying level of burnout [3], between 10% and 15% of Swiss and Finnish adolescents have a severe burnout level [17,18] and more than 40% of French adolescents have a high level of burnout [13]. As with work burnout, it seems that estimating the prevalence of school burnout is problematic given the absence of consensus on its diagnostic criteria [19]. Also, note that the concept of burnout is often questioned in the medical literature, especially through an ongoing debate on the burnout-depression overlap [19,20,21]. However, as the negative effects of school burnout on academic and cognitive performance seems to be above and beyond those associated with depression or anxiety [22], this concept remains relevant for addressing the effects of school stress on students.

In addition, a number of determinants of school burnout have already been identified within and outside the school context. External factors such as parents’ work-related burnout [23], learning difficulties [24] and pre-existing internalizing disorders [25] are known to be positively associated with school burnout. In addition, as observed in the case of academic stress [4,6], several studies have shown that the severity of school burnout varies by gender (girls are more at risk), school grades (students in pre- and post-transition grades are more at risk) and school tracks (student in academic tracks are more at risk) [26,27]. Adolescents who aim for achievement and academic performance have higher levels of burnout than those aiming at learning or mastering a subject [28]. It also appears that adolescents who are stressed due to academic demands and worried about their academic and/or professional future have particularly high levels of exhaustion and feelings of inadequacy [26]. Finally, transitions are critical moments for the onset or development of burnout, especially for adolescents who are studying in academic tracks [27]. Conversely, it would appear that a good school climate [29] and high self-efficacy beliefs [30] are associated with lower levels of school burnout.

These studies highlight school burnout as positively associated with certain demands related to school, and negatively associated with school and/or individual resources. As shown by Salmela-Aro and Upadyaya [30], application of the demand-resources model [31] is also relevant in the school context to understand the mechanisms that can lead to adolescent burnout. According to this model, school burnout would be the result of an effort-driven energetic process of overtaxing and wearing out in which excessive school demands outweigh available resources. Reciprocally, the availability of resources would support a motivational process promoting school engagement [30]. It therefore seems important to identify school demands as well as individual or school resources that may influence levels of adolescent burnout. In addition, in the continuity of work on professional burnout, a particular attention should be paid to situational factors (such as certain task characteristics or organizational factors) known as having a greater influence on burnout than individual factors [32]. In the school context, these characteristics or situational factors can be summarized under the concept of educational practices [33].

### 1.2. Educational Practices

As mentioned above, several school characteristics may influence students’ health. They can be described at several levels and involve teachers as well as other professionals in the school. Eccles and Roeser [1], for example, refer to different levels of school organization. One is centered on teachers, including their instructional behavior, motivation and the quality of their relationship with students. Another level has broader, school-wide characteristics. These include aspects related to school culture, such as the way problems are treated, hence, how rules are implemented and applied. School culture also encompasses collaboration between school and family and the opportunities students have of participating actively in school life. In the present paper, all these aspects will be referred to as educational practices.

Likewise, Janosz, Georges and Parent [33] defined educational practices as what teachers, management and the various professionals in the school do on a daily basis. In their attempt to describe the school environment and assess its influence on students’ adjustment and academic success, Janosz and Bouthillier [34] have defined which categories of educational practices are recognized by research and field professionals as influencing the quality of students’ behavior and learning. Eight of these were considered of particular interest. (1) Support for struggling students. This refers to the students’ perceptions of the support they might receive if they encounter academic or personal difficulties. (2) Teachers’ instructional behavior. This refers to strategies such as the use of cooperative pedagogies or taking into account pupils’ learning strategies, as well as to the flexibility of practices addressing the students’ various needs. (3) Teaching time, which refers to efficient lesson planning and the strategies that are applied to minimize the loss of time before and during the course. (4) Rule clarity and implementation, which refers the ease with which students can understand rules and the consequences of their violation, obtain details about these rules and the knowledge of the rules by students and other school members. (5) Application of rules, which refers to the ways in which rules are applied by teachers and principals (e.g., consequences of rules violation are applied as foreseen by regulations). (6) Students’ participation in school life, which refers to the possibility for students to give their opinions about how the school should work, participate in the choice of rules and take part in certain decisions. (7) Behavioral management, which refers to teachers’ motivation to teach and to maintain order and calm in the classroom. (8) School–family relationships, which refers to practices that promote communication between the school and the parents, their involvement in school life and the support they can receive to help their children with schoolwork and engagement. These dimensions can be measured with the School Socioeducational Environment Questionnaire, which is used to document the quality of certain characteristics of the school environment, providing the viewpoint of different actors in the school [34]. A version has been adapted to the context of a French-speaking region of Switzerland [35].

### 1.3. Educational Practices and School Burnout

As Janosz, Georges, & Parent [33] have pointed out, the above-mentioned practices are likely to influence certain elements of the school context. For example, a good educational climate is fostered when teaching practices are of good quality and there is not too much time wasted on the management of behavioral problems in the classroom. In addition, academic success is encouraged when teachers’ attitudes and expectations reflect a belief in the students’ ability to succeed. More recently, studies have also shown that certain teaching practices can act as protective factors against problems such as stress or school burnout.

On the one hand, a number of studies have highlighted the potentially protective effect of some teacher practices. For example, a high level of teacher support indirectly decreases the risk of burnout by reducing the level of school stress and, consequently, its pathological impact on students [36]. In another study among Finnish adolescents, Salmela-Aro et al. [29] have shown that the level of burnout was negatively predicted by teachers’ motivation. Indeed, the adolescents with the lowest burnout scores tended to be those who felt encouraged to share their opinions in class, who feel that their teachers took an interest in their work and that they were treated fairly. Conversely, school burnout was positively predicted by a negative class climate which, in the framework of this study [29], was a global reflection of a sense of restlessness and haste in the classroom. Furthermore, Pilkauskaite-Valickiene, Zukauskiene and Raiziene [37] specified this link by addressing school climate in a dimensional way. These authors showed that being able to easily express one’s opinion in class and teachers’ consideration for their point of view was associated with low scores of emotional exhaustion, cynicism and inadequacy towards school.

On the other hand, several authors have emphasized the importance of certain practices on the level of school structure and organization. For example, it seems that the risk of burnout is lower in schools where students feel they can find help among the various professionals available [29]. It also appears that some causes of burnout such as lack of control, lack of reward for one’s contribution or lack of equity are found in the school contexts in which students cannot participate in decisions about programs or teaching methods, success and learning are insufficiently valued, work is not assessed by means of grades, and/or students have a feeling of injustice with regard to the evaluation of their schoolwork [3].

### 1.4. Research Problem and Hypotheses

Overall, work on school burnout emphasizes the role of individual teaching practices as well as school-level factors pertaining to structure and functioning. However, these different levels of educational practices have generally been evaluated independently [29,36,37], which does not allow for the relative weights of their impact on school burnout to be calculated. Moreover, their effect was often shown on a global measure of school burnout [29]. A better knowledge of the impact of these practices on the dimensions of school burnout would make it possible to better target preventive interventions. The aim of this study is therefore to identify the practices that have the greatest influence on school burnout in adolescents and to specify their effects on its dimensions.

**Figure 1:**
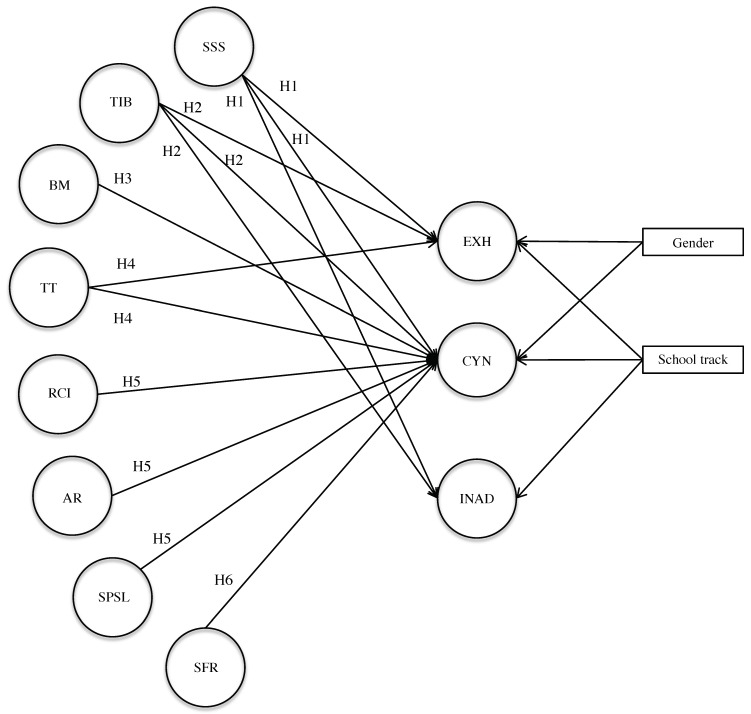
Hypothetical model (showing only latent variables). EXH = Exhaustion, CYN = Cynicism, INAD = Inadequacy; SSS = Support for Struggling Students, TIB = Teachers’ Instructional Behavior, TT = Teaching Time, RCI = Rule clarity and implementation, AR = Application of rules, SPSL = Students’ Participation in School Life, BM = behavioral management, SFR = School–family relationships.

Based on the above-mentioned theoretical considerations and empirical results, we constructed a hypothetical model of the relationships between school burnout dimensions and educational practices (see Figure 1). We first hypothesize (H1) that the support given to struggling or distressed students influences the level of the three dimensions of school burnout, in accordance with work showing that the risk of school burnout is less important when students have access to health professionals in schools [29]. Then, in the continuity of research stressing the importance of teacher attitudes [29,38], we assume that the dimension “teachers’ instructional behavior” influences the three dimensions of school burnout (H2). Following previous studies highlighting the protective effect of a positive classroom climate on students’ cynicism [37], we also expect behavioral management (which, to some extent, can also be considered as part of the classroom climate) to influence the cynicism aspect of burnout (H3). We also hypothesize (H4) that time devoted to teaching influences levels of exhaustion and cynicism because the time lost in class can foster a climate of haste, which effects on burnout have already been shown [29]. Then, since the way rules are established and applied influences the climate of justice [33] and that cynicism could be an inappropriate coping strategy when facing a sense of injustice [12,17], we assume that rule-related dimensions (i.e., rule clarity and implementation; application of rules) as well as student participation in school life influence the level of cynicism (H5). Indeed, by putting students in a “situation of passivity” (i.e., listening without speaking, submitting to instructions, etc.) at a time when they are trying to become more active in their environments, school demands may aggravate the problems related to adolescence [1]. Thus, a student who feels that he or she cannot decide on anything and that everything is imposed on him or her at school could defend himself or herself from this feeling by developing cynicism towards school. Finally, since a positive family–school relationship leads to better outcomes for students [39] and since parental engagement influences student motivation [40], we can hypothesize (H6) that family–school relations influence the cynicism dimension of school burnout.

## 2. Materials and Methods.

The present study was cross-sectional and conducted with high school students in the French-speaking part of Switzerland. Subjects were recruited from a single public high school that requested research to better identify the determinants of academic success among its students. Data were collected in 2018 by authors N.M., J.M. and M.R.

### 2.1. Ethical Statement

This study has been positively previewed by an inter-institutional research commission and authorized by the Direction Générale de l’Enseignement Post-obligatoire of the Vaud canton (ethic approval code: ID159). The school direction gave its authorization after being informed of the study procedure. Our research complies with the code of ethics of the Swiss Society of Psychology (SSP), including data anonymization and participants’ informed consent. For students under 18 years old, written informed consent was obtained from their parents (or legal guardians).

### 2.2. Subjects and Procedure

Initially, 372 adolescents were recruited from a public high school in French-speaking part of Switzerland. Theses adolescents attend their first year of post-compulsory academic schooling. They responded collectively and in the presence of a research assistant to a questionnaire with several scales. Search for outliers (e.g., same response to all items) indicated a need to delete data from 12 subjects. After listwise deletion of missing values, our sample consists of 287 adolescents (146 girls) aged between 14 and 19 years. old (*M* = 16.08, *SD* = 1.01). The adolescents come from diverse sociocultural backgrounds in situations where both parents have a higher education (36.5%), only one parent has a higher education (31.1%) and none of the parents have a higher education (32.4%).

### 2.3. Measures

#### 2.3.1. School Burnout

A French version [38] of the School Burnout Inventory (SBI) [16] was used to measure school burnout. This questionnaire consists of 9 items on burnout related to school demands (e.g., “I feel overwhelmed by my schoolwork”), cynicism about school (e.g., “I am constantly wondering if my school work makes sense”) and the feeling of inadequacy as a student (e.g., “I often feel that I am insufficient in my school work”). The SBI items are scored on a six-point Likert scale ranging from “Completely False” to “Completely True” and provide a total score as well as dimensional scores. A high score indicates that the student is at risk on a given dimension.

#### 2.3.2. Educational Practices

Educational practices were measured using the School Socioeducational Environment Questionnaire (SSEQ) developed by Janosz, Georges, & Parent [33,34] and adapted to the context of French-speaking part of Switzerland by Sangsue and Vorpe [35]. This instrument contains 62 items that focus on school climate, school problems and educational practices. As part of our study, we only focus on 38 items related to eight educational practice dimensions (see Appendix A). These were the following: support for struggling students (3 items, e.g., “When students have problems in school, it is easy for them to get help from adults in this school.”); teachers’ instructional behaviors (5 items, e.g., “Teachers encourage students to do their best.”); teaching time (5 items, e.g., “During classes, we waste a lot of time because of disturbing students.”); rule clarity and implementation (5 items, e.g., “Rules are clear and easy to understand.”); application of rules (4 items, e.g., “Teachers enforce the rules as prescribed in the school regulations.”); student participation in school life (4 items, e.g., “Students are ask for their opinion on how school works”); behavioral management (9 items, e.g., “It is rare to see a teacher shouting at a student in front of the whole class.”); school–family relationships (3 items, e.g., “Parents have their place in this school.”). For each item, students were asked to express their degree of agreement or disagreement on a six-point Likert scale ranging from “totally disagree” to “strongly agree”. For some items, scores have been inverted. Consequently, for each item, the higher the score, the more students feel that educational practices are “positive”.

#### 2.3.3. Variables Related to Personal and School Contexts

Various sociodemographic and school-related data were collected for control purposes. Among these, we retained gender and school track, for which effects on school burnout have already been shown [7,26].

### 2.4. Statistical Analysis

First, confirmatory factor analyses were performed using Analysis of Moment Structures (AMOS), (IBM SPSS, Chicago, IL, USA) [41] in order to test the structure of the School Burnout Inventory as well as the scale of Educational Practices. Next, we examined the reliability of our scales as well as the mean value and distribution of scores for each of the main variables in our study. Then, partial correlation analyses were carried out by controlling the effects of age, gender and school track, indicate the relationships between perceived educational practices and school burnout. Finally, we verified our hypotheses by testing our theoretical model with path analysis.

The models’ goodness of fit is evaluated using the following indices [42]: chi-square; relative chi-square (CMIN/DF) of three or less; Root Mean Square Error of Approximation (RMSEA) and Standardized Root Mean Square Residual (SRMR) of 0.08 or less; Comparative Fit Index (CFI) and Tucker-Lewis Index (TLI) of 0.90 or above and Akaike Information Criterion (AIC) whose lowest value indicates which model best fits the data. Gender, school track and age were assigned as controlled variables.

## 3. Results

### 3.1. Preliminary Analysis

With regard to school burnout scale, results of the confirmatory analysis give relatively satisfactory indices, *χ*^2^ (24, *N* = 272) = 75.45, *p* < 0.001, *χ*^2^/*df* = 3.14, RMSEA = 0.09, CFI = 0.94, TLI = 0.91, SRMR = 0.06, AIC = 135.45. These results are in line with what was expected, namely that school burnout can be approached as a tree-dimensional construct composed of Exhaustion, Cynicism and Inadequacy.

Regarding the scale of educational practices, the results of the confirmatory factor analyses yield unsatisfactory indices, *χ*^2^ (629, *N* = 266) = 1278.80, *p* < 0.001, *χ*^2^/*df* = 2.03, RMSEA = 0.06, CFI = 0.85, TLI = 0.82, SRMR = 0.08, AIC = 1578.79. While examining the original validation study [34], we noticed that four items subsequently identified as problematic (three related to Behavioral Management and one related to Teaching Time) were present in the adaptation for the French speaking part of Switzerland [35]. These items were therefore removed. In addition, regression weights of some items related to Behavioral Management were problematic. When looking more closely to the individual items of this latter dimension, two of them were clearly more related to teachers’ motivation than to behavioral management per se. The consideration of these two items as a separate dimension (Teacher Motivation), yielded a clear improvement of the adjustment indices: *χ*^2^ (491, *N* = 266) = 753.61, *p* < 0.001, *χ*^2^/*df* = 1.53, RMSEA = 0.05, CFI = 0.93, TLI = 0.91, SRMR = 0.06, AIC = 1029.61. After these changes, educational practices therefore had nine dimensions (instead of eight) and its original Behavioral Management dimension was reduced to four remaining items.

Regarding the reliability of the scales used, Cronbach’s alpha coefficients are reported in Table 1. Most of the values are between 0.74 and 0.86, indicating satisfactory to good internal consistency [43]. However, values are lower for the Inadequacy (α = 0.55) and Behavioral Management (α = 0.57) dimensions, indicating poor internal consistency.

### 3.2. Descriptive Analysis

Table 1 also includes distribution coefficients, means and standard deviations as well as correlation coefficients for School Burnout scores and Educational Practices. First, results show that most skewness and kurtosis coefficients are within the ± 1 range recommended by Muthén and Kaplan [44], and none exceed the critical threshold of ± 3 [45]. Then, it appears that the Inadequacy dimension predominates (*M* = 3.31, *SD* = 1.26) in relation to Exhaustion (*M* = 2.84, *SD* = 1.14) and cynicism (*M* = 2.81, *SD* = 1.43), which have relatively similar scores. Regarding educational practices, it appears that the dimensions Rule Clarity and Rule Implementation (*M* = 4.60, *SD* = 0.94) as well as Application of Rules (*M* = 4.77, *SD* = 0.80) obtained the highest scores. On the other hand, students’ Participation in School Life is the lowest score (*M* = 2.99, *SD* = 1.11). Partial correlational analyses (Table 1) show that most dimensions related to educational practices are significantly and negatively correlated with the Cynicism dimension of burnout, with *r* values ranging from −0.15 (Application of Rules) to −0.36 (Teacher Motivation). In addition, almost half of the educational practices are also negatively correlated with Exhaustion (*r* from −0.14 for Instructional Behaviors to −0.21 for Support for Students). However, Application of Rules is the only educational practice to be slightly negatively correlated (*r* = −0.14) with the Inadequacy dimension of burnout.

### 3.3. Path Analysis

Initial analysis shows that the adjustment of the hypothetical model is relatively satisfactory, *χ*^2^ (927, *N* = 266) = 1322.05, *p* < 0.001, *χ*^2^/*df* = 1.42, RMSEA = 0.04, CFI = 0.92, TLI = 0.92, SRMR = 0.06, AIC = 1722.05. However, it appears that (1) Support for Struggling Students, Teaching Time, Rule Clarity and Implementation, Application of Rules, student Participation in School Life, Behavioral Management and School–family Relationships are not significantly associated with Cynicism; (2) Teachers’ Instructional Behavior are not significantly associated with school burnout and (3) Support for Struggling Students, Teachers’ Instructional Behavior, and Teacher Motivation are not significantly associated with Inadequacy. We revised the hypothetical model by removing these relationships as well as the variables no longer directly associated with a dimension of school burnout. On the basis of modification indices, we also added a path between Application of Rules and Inadequacy. The final model fit indices are satisfactory, *χ*^2^ (331, *N* = 266) = 460.09, *p* < 0.001, *χ*^2^/*df* = 1.39, RMSEA = 0.04, CFI = 0.96, TLI = 0.95, SRMR = 0.06, AIC = 666.09. In addition, comparison of AIC values using standard error estimation (*SE*), calculated by the Bootstrap method [41,46], shows a significant improvement in the fit between the hypothetical model (*SE* = 2406.07, *SD* = 4.70) and the final model (*SE* = 836.48, *SD* = 2.22).

**Figure 2:**
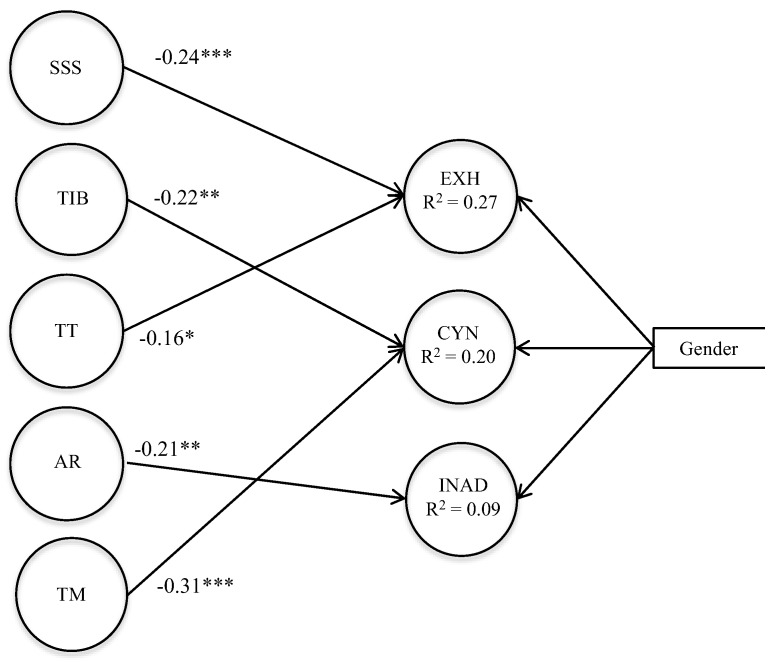
Estimated final model with predictors (only statistically significant regression coefficients are given). SSS = Support for Struggling Students, TIB = Teachers’ Instructional Behavior, TT = Teaching Time, AR = Application of rules, TM = Teachers’ motivation; EXH = Exhaustion, CYN = Cynicism, INAD = Inadequacy; * *p* < 0.05, ** *p* < 0.01, *** *p* < 0.001.

The final model is presented in Figure 2. It first appears that only five educational practices are significantly associated with school burnout. In particular, our results highlight that Support for Struggling Students as well as Teaching Time are negative predictors of Exhaustion (ß = −0.24, *p* < 0.001; ß = −0.16, *p* < 0.05, respectively). Then, it appears that Application of Rules is a direct and negative predictor of Inadequacy (ß = −0.21, *p* < 0.01). Finally, we observe that Teacher Motivation as well as Teachers’ Instructional Behavior are negative predictors of Cynicism (ß = −0.31, *p* < 0.001; ß = −0.22, *p* < 0.01, respectively). Taken together, the predictors explained 27% of the variance for Exhaustion, 20% for Cynicism and 9% for Inadequacy.

## 4. Discussion

The final structural model shows that support for students facing difficulties in school only influences their reported levels of exhaustion, which partially confirms our hypothesis regarding the protective effect of this educational practice on the three dimensions of school burnout (H1). Thus, in the continuity of work showing that the risk of school burnout is lower when students have access to health professionals in schools [29], our results suggest that it is the level of exhaustion that diminishes when students feel that they can benefit from support in case of academic and/or personal difficulties. Our hypothesis H2 is also partially confirmed since teachers’ instructional behavior only influences the level of students’ cynicism. These results allow us to specify the protective role of certain teacher practices with regard to school burnout [29,36] by showing that students are less likely to develop a cynical attitude toward school when they perceive that their teachers are trying to motivate them, value their efforts and achievements or make sure they have understood one subject before moving on to the next. Our hypothesis H3 was partially confirmed. Indeed, as suggested by prior work, how teachers maintain order in the classroom and their motivation to teach should be considered as two separate dimensions of behavioral management. Accordingly, our final model only showed a negative effect of teacher motivation on the level of cynicism. As students’ cynicism includes some motivational aspects (i.e., lack of motivation toward schoolwork), it is not so surprising to find such a negative effect since teachers’ motivation has previously been shown to influence students’ motivation [47,48]. One could also argue that motivated teachers will develop higher quality activities likely to help students find meaning and interest in their schoolwork, thus promoting increased engagement on their part [49]. Behavioral management does not seem to impact cynicism significantly. In other words, the interest and meaning that students associate with their schoolwork does not seem to be influenced by the way the teachers react to disruptive classroom behavior. Hypothesis H4 was partially confirmed in that the time devoted to teaching has an influence on Exhaustion but not on Cynicism. One possible interpretation could be that students who feel they are wasting time in class may compensate for time loss outside the classroom, hence increasing their work load. This would explain more precisely previous research reporting that a negative classroom climate, including feelings of haste, is a risk factor for school burnout [29]. In contrast, our results do not confirm our hypothesis H5 on cynicism as a way of dealing respectively with a feeling of injustice related to the way that rules are managed within the school [33]. This could be explained by the fact that we measured a form of cynicism that is a reflection of indifference and a loss of meaning with regard to schoolwork rather than the expression of critical attitudes towards school and its functioning. Thus, even if students feel that the rules are unclear, ill-implemented and/or unfairly applied, or if they cannot participate enough in decisions about the school’s functioning, this does not seem to affect the interest and meaning they place in their schoolwork. We also find that application of rules was negatively related to feelings of inadequacy. One interpretation could be that a proper application of rules would prevent the feeling of injustice in students, which is known (at least in adults) to be a risk factor for burnout [50]. It is also worth mentioning that the application of rules has the particularity of being present at both school- and teacher-levels, which stresses the importance of cohesion between these two levels. Finally, our results also invalidate our hypothesis H6 regarding the effect of school–family relationships on the level of cynicism. Here it should be noted that in Switzerland, students within the age range and academic track that are included in this study attend particular schools (called “gymnases” in French) in which school–family relationships are restricted essentially to communicating grades to parents when students are under 18 years old. Therefore, it could be argued that this dimension does not play an important role in such a student population.

This study also has a number of limitations. First, care should be taken with regard to the generalization of results, since all students attend the same school, which, as described above, represents academic track with particular characteristics. A larger sample comprising students from a diverse array of French-speaking Swiss schools would allow to control school-level effects (in particular were the distinction between academic and vocational tracks is concerned). Indeed, even though Salmela-Aro et al. [29] have shown that school burnout scores differ only slightly between schools, effects of contextual moderators of stress and burnout may be better captured using multilevel models at the school and/or class level [5]. Next, it is important to emphasize that causality cannot be directly tested in this study because of its cross-sectional nature. Indeed, based on the associations that we have highlighted, we can assume that a high level of support for struggling students could reduce the level of exhaustion, but also that a high level of exhaustion could worsen the students’ perception of the support available. Longitudinal studies would give a better understanding of the causal links between perceived educational practices and school burnout. It should also be noted that the use of self-questionnaires involves methodological bias [51] and allows only for a subjective assessment of educational practices and school burnout. Some of the associations we have highlighted could therefore be also explained by other variables. For example, in line with the work that has highlighted the overlapping problems between burnout and depression [19,20,21], we can assume that a student with depressive symptoms reports more exhaustion and that this exhaustion leads him or her to see the school context as less supportive. Future work on school burnout should therefore more systematically control certain variables such as depression. Also, subsequent studies could benefit from the use of subjective and objective measures, particularly with regard to the observation of those educational practices that are actually implemented in a school. Finally, the internal consistency (Cronbach’s alpha) of the Inadequacy dimension of the school burnout inventory and the Behavioral Management dimension of the scale of educational practices is relatively low. Regarding Inadequacy, this problem has already been raised in other studies that have used the Lithuanian [37], French [38,52], or Spanish [53] versions of the School Burnout Inventory. The small number of items composing this dimension could partly explain these results [38], as well as difficulties understanding the exact meaning of certain items. Regarding Behavioral Management, we have no point of comparison since this scale was modified on the basis of our preliminary results. Future work could seek to enhance the psychometric qualities of these scales by reformulating and/or adding items.

## 5. Conclusions

The aim of this study was to identify the educational practices that have the greatest influence on school burnout in high school adolescents and to specify their effects on its three dimensions. We found that some teacher- and school related educational practices are negatively and specifically associated with school burnout dimensions. Indeed, support for struggling students and teaching time are associated with exhaustion; teachers’ instructional behavior and teacher motivation are associated with cynicism; and application of rule is associated with inadequacy. Thus, these practices should be of particular interest when it comes to strengthening the protective role of schools and teachers against school burnout in adolescents.

## Figures and Tables

**Table 1:** Reliability, descriptive statistics and partial correlations of school burnout and educational practices scores.

	Cronbach’s α	M	SD	Skewness	Kurtosis	1	2	3
1. Exhaustion	0.74	2.83	1.1	0.4	−0.55	-	0.36 **	0.41 ***
2. Cynicism	0.85	2.82	1.36	0.65	−0.43	0.36 ***	-	0.47 ***
3. Inadequacy	0.55	3.30	1.24	0.17	−0.71	0.41 ***	0.47 **	-
4. Support for students	0.81	4.60	0.93	−0.92	1.01	−0.21 ***	−0.17 **	−0.11
5. Teachers’ instructional behavior	0.82	4.79	0.85	−0.88	1.29	−0.14 *	−0.33 ***	−0.12
6. Teaching time	0.74	2.97	1.1	0.2	−0.47	−0.15 *	−0.11	−0.07
7. Rule clarity and implementation	0.86	3.41	1.2	−0.08	−0.62	−0.01	−0.16 **	−0.07
8. Application of rules	0.79	3.64	0.92	−0.17	−0.33	−0.09	−0.15 *	−0.14 *
9. Student participation	0.86	4.69	1.09	−0.83	0.47	−0.07	−0.18 **	−0.03
10. Behavioral management	0.57	3.77	1.1	−0.43	−0.22	−0.12	−0.22 ***	−0.06
11. School–family relationships	0.77	3.68	1.18	−0.25	−0.65	−0.01	−0.11	−0.09
12. Teacher motivation	0.81	3.84	1.31	−0.13	−0.82	−0.17 **	−0.36 ***	−0.06

Note. * *p* < 0.05, ** *p* < 0.01, *** *p* < 0.001.

## Appendix A

French version of the School Socioeducational Environment Questionnaire [35] was kindly provided to us by Dr. Sangsue and is largely based on the original version of Janosz, Georges, & Parent [33] with minor adaptations to the French-speaking Swiss context. It originally contained 42 questions on educational practices. We discarded one item related to school–family relationships as well as three items related to the dimension of "oversight" because they did not apply to the context of our high school students. Our final questionnaire therefore contained 38 items.

## References

[1] Eccles J.S., Roeser R.W. (2011). Schools as developmental contexts during adolescence. J. Res. Adolescence.

[2] Rutter M. (1991). Pathways from childhood to adult Life; the role of schooling. Pastor. Care Educ..

[3] Slivar B. (2001). The syndrome of burnout, self-image, and anxiety with grammar school students. Horizon Psychol..

[4] Kouzma N.M., Kennedy G.A. (2004). Self-reported sources of stress in senior high school students. Psychol. Rep..

[5] Torsheim T., Wold B. (2001). School-related stress, support, and subjective health complaints among early adolescents: A multilevel approach. J. Adolescence.

[6] Banks J., Smyth E. (2015). ‘Your whole life depends on it’: Academic stress and high-stakes testing in Ireland. J. Youth Stud..

[7] Walburg V. (2014). Burnout among high school students: A literature review. Child. Youth Serv. Rev..

[8] Collishaw S. (2015). Annual research review: Secular trends in child and adolescent mental health. J. Child. Psychol. Psyc..

[9] Eichenberger Y., Kretschmann A., Delgrande Jordan M. (2017). Stress Lié au Travail Scolaire chez les Adolescent-e-s en Suisse: Chiffresactuels, évolution au Cours du Temps et Bien-être des Adolescent-e-s Concerné-e-s.

[10] Sweeting H., West P., Young R., Der G. (2010). Can we explain increases in young people’s psychological distress over time?. Soc. Sci. Med..

[11] Bask M., Salmela-Aro K. (2013). Burned out to drop out: exploring the relationship between school burnout and school dropout. Eur. J. Psychol. Educ..

[12] Parker P.D., Salmela-Aro K. (2011). Developmental processes in school burnout: A comparison of major developmental models. Learn Individ. Differ..

[13] Zakari S., Walburg V., Chabrol H. (2008). Étude du phénomène d’épuisement scolaire, de la dépression et des idées de suicides chez des lycéens français. J. Ther. Comport. Cogn..

[14] Pedrini L., Sisti D., Tiberti A., Preti A., Fabiani M., Ferraresi L., Palazzi S., Parisi R., Ricciutello C., Rocchi M.B.L. (2015). Reasons and pathways of first-time consultations at child and adolescent mental health services: an observational study in Italy. Child. Adol. Psych. Men.

[15] Genoud P.A., Brodard F., Reicherts M. (2009). Facteurs de stress et burnout chez les enseignants de l’écoleprimaire. Eur. Rev. Appl. Psychol..

[16] Salmela-Aro K., Kiuru N., Leskinen E., Nurmi J.E. (2009). School Burnout Inventory: reliability and validity. Eur. J. Psychol. Assess..

[17] Meylan N. (2015). Le Burnout des élèves: Validation du School Burnout Inventory, Identification de Facteurs de Risque et de Protection et Exploration des Liens avec la Consommation de Substances. Thèse de Doctorat.

[18] Salmela-Aro K., Tynkkynen L. (2012). Gendered pathways in school burnout among adolescents. J. Adolescence.

[19] Bianchi R., Schonfeld I.S., Laurent E. (2015). Burnout–depression overlap: A review. Clin. Psychol. Rev..

[20] Schonfeld I.S., Bianchi R., Palazzi S. (2018). What is the difference between depression and burnout? An ongoing debate. Riv. Psichiatr..

[21] Bianchi R., Verkuilen J., Brisson R., Schonfeld I.S., Laurenet E. (2016). Burnout and depression: Label-related stigma, help-seeking, and syndrome overlap. Psychiatry Res..

[22] May R.W., Bauer K., Fincham F.D. (2015). School burnout: diminished academic and cognitive performance. Learn Individ. Differ..

[23] Salmela-Aro K., Tynkkynen L., Vuori J. (2011). Parents’ work burnout and adolescents’ school burnout: Are they shared?. Eur. J. Dev. Psychol..

[24] Kiuru N., Leskinen E., Nurmi J.E., Salmela-Aro K. (2011). Depressive symptoms during adolescence: Do learning difficulties matter?. Int. J. Behav. Dev..

[25] Salmela-Aro K., Savolainen H., Holopainen L. (2009). Depressive symptoms and school burnout during adolescence: Evidence from two cross-lagged longitudinal studies. J. Youth Adolesc..

[26] Meylan N., Doudin P.A., Curchod D., Stephan P. (2011). School burnout in adolescents: Differences in background variables and exploration of school-related stress at the end of compulsory schooling. Ric. Psychol..

[27] Salmela-Aro K., Kiuru N., Nurmi J.E. (2008). The role of educational track in adolescents’ school burnout: A longitudinal study. Br. J. Educ. Psychol..

[28] Tuominen-Soini H., Salmela-Aro K., Niemivirta M. (2008). Achievement goal orientations and well-being. Learn Instr..

[29] Salmela-Aro K., Kiuru N., Pietikäinen M., Jokela J. Does school matter? The role of school context for school burnout. Eur. Psychol..

[30] Salmela-Aro K., Upadyaya K. (2014). School burnout and engagement in the context of demands, resources model. Br. J. Educ. Psychol..

[31] Bakker A., Demerouti E. (2007). The job demands-resources model: State of the art. J. Manag. Psychol..

[32] Maslach C., Schaufeli W.B., Leiter M.P. (2001). Job burnout. Annu. Rev. Psychol..

[33] Janosz M., Georges P., Parent S. (1998). L’environnement socioéducatif à l’école secondaire: Un modèle théorique pour guider l’évaluation du milieu. Rev. Psychoeduc..

[34] Janosz M., Bouthillier C. (2007). Rapport de Validation du Questionnaire sur L’Environnement Socioéducatif des écoles Secondaires (QES-secondaire).

[35] Sangsue J., Vorpe G. (2004). Influences professionnelles et personnelles du climat scolaire chez les enseignants et les élèves. Psychol. Trav. Organ..

[36] Meylan N., Doudin P.A., Curchod-Ruedi D., Stephan P. (2015). School burnout and social support: the importance of parent and teacher support. Psychol Fr..

[37] Pilkauskaite-Valickiene R., Zukauskiene R., Raiziene S. (2011). The role of attachment to school and open classroom climate for discussion on adolescent’s school-related burnout. Procedia Soc. Behv..

[38] Meylan N., Doudin P.A., Antonietti J.P., Stephan P. (2015). School Burnout Inventory: A French validation. Eur. Rew. Appl. Psychol..

[39] Thompson G.L. (2003). No parent left behind: Strengthening ties between educators and African American parents/guardians. Urban Rev..

[40] Pomerantz E.M., Moorman E.A., Litwack S.D. (2007). The how, whom, and why of parents’ involvement in children’s academic lives: More is not always better. Rev. Educ. Res..

[41] Arbuckle J.L. (2012). Amos User’s Guide Version 21.

[42] Hu L., Bentler P.M. (1998). Fit indices in covariance structure modeling: Sensitivity to underparameterized model misspecification. Psychol. Methods.

[43] DeVellis R.F. (1991). Scale Development—Theory and Applications.

[44] Muthén B., Kaplan D.A. (1985). Comparison of Some Methodologies for the Factor Analysis of Non-normal Likert Variables. Brit. J. Math. Stat. Psy..

[45] Kline R.B. (1998). Principles and Practice of Structural Equation Modelling.

[46] Bollen K.A., Stine R.A. (1992). Bootstrapping goodness-of-fit measures in structural equation models. Sociol. Methods Res..

[47] Atkinson E.S. (2000). An investigation into the relationship between teacher motivation and pupil motivation. Educ. Psychol..

[48] Roth G., Assor A., Kanat-Maymon Y., Kaplan H. (2007). Autonomous motivation for teaching: How self-determined teaching may lead to self-determined learning. J. Educ. Psychol..

[49] Fredricks J.A., Blumenfeld P.C., Paris A.H. (2004). School engagement: Potential of the concept, state of the evidence. Rev. Educ. Res..

[50] Bria M., Baban A., Dumitrascu D.L. (2012). Systematic review of burnout risk factors among European healthcare professionals. Cogn. Brain Behav..

[51] Razavi T. (2001). Self-Report Measures: An Overview of Concerns and Limitations of Questionnaire use in Occupational Stress Research.

[52] Oller-Perret C., Walburg V. (2018). Impact of school-related burnout on alcohol consumption behavior among adolescents. Psychol. Fr..

[53] Moyano N., Riano-Hernandez D. (2013). Burnout escolar en adolescents espanoles: Adaptacion y validation del School Burnout Inventory. Ansiedad y Estrés.

